# Kirigami Patterning of MXene/Bacterial Cellulose Composite Paper for All‐Solid‐State Stretchable Micro‐Supercapacitor Arrays

**DOI:** 10.1002/advs.201900529

**Published:** 2019-04-12

**Authors:** Shangqing Jiao, Aiguo Zhou, Mingzai Wu, Haibo Hu

**Affiliations:** ^1^ School of Physics and Materials Science Anhui University Hefei 230601 China; ^2^ School of Materials Science and Engineering Henan Polytechnic University Jiaozuo Henan 454000 China

**Keywords:** bacterial cellulose, kirigami, micro‐supercapacitor arrays, MXene, stretchable

## Abstract

Stretchable micropower sources with high energy density and stability under repeated tensile deformation are key components of flexible/wearable microelectronics. Herein, through the combination of strain engineering and modulation of the interlayer spacing, freestanding and lightweight MXene/bacterial cellulose (BC) composite papers with excellent mechanical stability and a high electrochemical performance are first designed and prepared via a facile all‐solution‐based paper‐making process. Following a simple laser‐cutting kirigami patterning process, bendable, twistable, and stretchable all‐solid‐state micro‐supercapacitor arrays (MSCAs) are further fabricated. As expected, benefiting from the high‐performance MXene/BC composite electrodes and rational sectional structural design, the resulting kirigami MSCAs exhibit a high areal capacitance of 111.5 mF cm^−2^, and are stable upon stretching of up to 100% elongation, and in bent or twisted states. The demonstrated combination of an all‐solution‐based MXene/BC composite paper‐making method and an easily manipulated laser‐cutting kirigami patterning technique enables the fabrication of MXene‐based deformable all‐solid‐state planar MSCAs in a simple and efficient manner while achieving excellent areal performance metrics and high stretchability, making them promising micropower sources that are compatible with flexible/wearable microelectronics.

## 1 Introduction

Recently, with the continuous accumulation of nanotechnology power, there has been a trend toward flexible/stretchable electronic devices with mechanically unconventional forms, such as sensory skins for robotics, flexible/bendable displays, and smart clothes, which represent novel applications that are impossible to realize with conventional electronics based on hard and brittle wafers.^1^, ^2^, ^3^, ^4^, ^5^, ^6^, ^7^, ^8^, ^9^, ^10^ An equally deformable micropower source is an indispensable component in the development and construction of these power‐independent flexible/stretchable electronic systems.^11^, ^12^, ^13^, ^14^, ^15^ Unfortunately, conventionally dominant energy storage systems such as Li‐ion batteries and capacitors, are usually stiff and heavy due to the use of slurry casting–based fabrication methods and rigid components.^16^, ^17^, ^18^, ^19^ Thus, there is an obvious mismatch in the mechanics and form between the traditional rigid energy storage devices and emerging deformable electronic devices. In this context, the development of equally deformable high‐performance energy storage systems that can overcome the existing mismatch is one of the key challenges for flexible/stretchable electronic devices.^20^, ^21^, ^22^, ^23^, ^24^

To date, a variety of novel deformable energy storage systems have been demonstrated for application as compatible power sources in flexible/stretchable electronic devices.^25^, ^26^, ^27^, ^28^, ^29^ Among them, by virtue of their high power density, short recharging time, long durability, safety, and simple structure, stretchable supercapacitors (SCs) have shown great potential as compatible deformable power sources.^30^, ^31^, ^32^, ^33^ Conventional stretchable SCs consisting of sandwich electrodes usually acquire their stretchability based on the prestrain applied to an elastic substrate before assembly of the components and the stretchability of the formed wave‐like electrodes bonded to the prestrained elastic substrate. In spite of their attractive features, such as high areal capacitance and simple fabrication process, stretchable SCs with sandwich electrodes configured in wave‐like shape usually cannot be easily integrated into planar stretchable electronic circuits. Additionally, the components configured in a wave‐like shape can be easily detached from each other under repeated tensile deformation, resulting in deterioration of the electrochemical performance. In contrast, the emerging 2D planar stretchable micro‐supercapacitor arrays (MSCAs) would be a better choice for integration into the usually planar stretchable electronic circuits due to their more rational segmented structural design:^34^, ^35^, ^36^ First, the micro‐supercapacitor units (islands) with a planar electrode configuration only serve as the active region for energy storage and do not involve tensile deformation, enabling the interdigital electrodes to maintain a constant distance and not to be destroyed during repeated tensile deformation, and thus guaranteeing the electrochemical stability of the device. Second, the 2D planar interdigital electrode configuration of the MSC islands can reduce the ionic diffusion pathway, resulting in a high power density for the device. Third, the elastic/stretchable conductive framework (bridges) interconnecting these MSC islands ensure the highly stretchable characteristics of the device. More importantly, these MSC islands can be arbitrarily grouped and interconnected by elastic/stretchable bridges, enabling on‐demand control of the output voltage and current density to meet actual requirements. Although impressive progress has been made, deformable planar MSCAs are still in the early stages of development and face several problems, such as a low production efficiency attributed to photolithography being the dominant method for fabricating the electrodes, the tedious complexity of the assembly process stemming from the complex island–bridge interconnected design, and more critically, the low areal capacitance, which is mainly caused by the traditionally used carbon material with a low specific capacitance.^37^, ^38^, ^39^ Thus, developing a simple and efficient approach to fabricate deformable planar MSCAs with a high areal capacitance, excellent stretchability, good integration capability, and electrochemical stability is still highly demanded but greatly challenging.

Generally, the areal capacitance of an MSC device is proportional to the specific capacitance of the used electrode materials.^40^, ^41^, ^42^ Employing a new electrode material with a high specific capacitance to design and fabricate the MSC islands is the most direct and effective method to improve the poor areal capacitance of MSCAs based on conventional carbon materials. Recently, a new type of energy storage material, namely, MXenes, has been developed.^43^, ^44^, ^45^, ^46^, ^47^, ^48^, ^49^, ^50^ Due to their unique 2D layered structure and metallic conductivity, MXenes exhibit a high specific volumetric capacitance, which offers great potential for improving the areal performance metrics of planar MSCs.^51^, ^52^, ^53^, ^54^, ^55^ However, few works on MXene‐based stretchable MSCAs with both high areal performance metrics and good elongation have been reportedly available so far. In addition, the restacking of the MXene nanosheets in the process of preparing the electrode films hinders the full penetration of the electrolyte and reduces the utilization efficiency of the active material, resulting in an underdeveloped areal capacitance of the subsequently fabricated device. To address this issue, 1D carbon nanofibers, such as carbon nanotubes, have been widely employed as interlayer nanospacers to alleviate the restacking of the 2D MXene sheets, leading to the improved performance of the composite films.^56^, ^57^, ^58^, ^59^, ^60^ However, the relatively high price, low yield of these carbon materials, and the need for strict control of their synthetic environment greatly restrict their large‐scale commercial application in the future. In contrast, cellulosic fibers have the advantages of being environmentally safe, renewable, abundant, and a sustainable natural resource.^61^ As one of these cellulosic fibers, bacterial cellulose (BC) nanofibers can be prepared entirely from pure natural substances without the use of expensive equipments, toxic chemical reagents, and special synthetic environments. Thus, they have the features of being low‐cost, abundant, easy to prepare, and environmentally friendly. Based on a consideration of the recycling economy and green chemistry, these low‐cost and renewable 1D BC nanofibers are promising candidates, serving not only as interlayer nanospacers to further improve the electrochemical performance of flexible MXene‐based electrode films, but also as a reinforcement material to enhance the mechanical properties of flexible MXene‐based electrode films to produce MXene‐based stretchable MSCAs with both high areal performance metrics and good elongation.

In this study, aimed at addressing the abovementioned challenges, we first designed a composite‐structured MXene paper based on 2D fully delaminated few‐layered Ti_3_C_2_T*_x_* MXene flakes and 1D bacterial cellulose fibers, which was fabricated via a facile all‐solution‐based paper‐making process. The 2D few‐layered MXene flakes were aligned along the planar direction, which could maximize the overlapping area and decrease the contact resistance. Additionally, the 1D BC fibers could serve as a stabilizer to link the 2D MXene flakes in the composite paper, dramatically increasing the mechanical strength of the hybrid paper. More importantly, the BC, with a 1D nanofiber structure, could serve as an interspacer to reduce the restacking of the fully delaminated few‐layered MXene flakes and modulate the interlayer spacing during paper production, facilitating ion transportation in the interlayer space and increasing the exposed amount of active material available for reaction. Thus, through the combination of strain engineering and modulation of the interlayer spacing, the freestanding and lightweight MXene/BC composite papers exhibited excellent mechanical stability and electrochemical performance. Then, with the aid of a simple, fast, and efficient laser‐cutting kirigami patterning technique, stretchable, bendable, and twistable all‐solid‐state planar MSCAs were acquired with a high areal capacitance of 111.5 mF cm^−2^ and no obvious degradation under tensile strain of up to 100% or when in bent or twisted states. The demonstrated combination of the high‐performance MXene/BC papers preparation method and the laser‐cutting kirigami patterning technique can simultaneously address the critical limitations regarding the “fabrication” and “performance” of stretchable MSCAs: 1) the all‐solution‐based preparation method accompanied by strain engineering and modulation of the interlayer spacing enables the low‐cost scalable production of MXene‐based paper electrodes with excellent mechanical strength and electrochemical performance for future MSCA devices; 2) the kirigami patterning process employing only a simple laser‐cutting technique does not involve any complex or cumbersome procedures and provides a single‐step process for preparing the segmented structure, greatly simplifying the assembly process and guaranteeing a low production cost and efficiency; 3) rational geometric design of the kirigami pattern allows the MSCA devices to be extremely stretchable, bendable, and twistable without obvious deterioration in their electrochemical performance, and more importantly, enables the MSC islands to be arbitrarily grouped and interconnected by a stretchable conductive framework, realizing on‐demand control of the output voltage and current density to meet actual requirements. All these results make the demonstrated protocol a powerful strategy for the development of MXene‐based planar MSCAs with high areal performance metrics, excellent stretchability, and good electrochemical stability for use as deformable micropower sources.

## 2 Results and Discussion

### 2.1 MXene/BC Composite Papers: Preparation, Strain Engineering, and Modulation of the Interlayer Spacing

**Figure 1:**
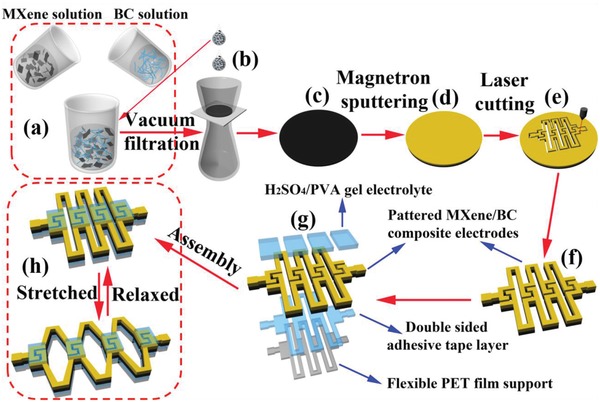
Flowchart of the protocol for preparing MXene/BC composite papers and their kirigami patterning with laser cutting for all‐solid‐state stretchable MSCAs. a) MXene/BC mixed solution via b) vacuum‐assisted self‐assembly to form the c) MXene/BC composite papers; d) magnetron sputtering of a Au layer on the as‐obtained MXene/BC composite paper; e) laser cutting to fabricate the f) patterned MXene/BC composite electrodes; g) assembled and exploded view of the fabricated MSCAs based on the patterned MXene/BC composite electrodes; h) schematic illustration of the stretched and relaxed MSCAs.

**Figure 2:**
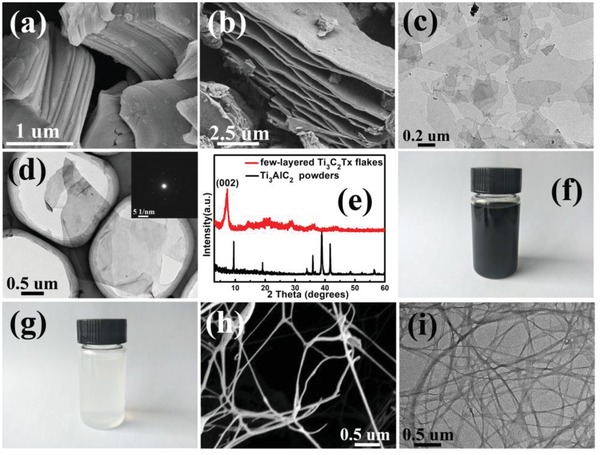
Representative SEM images of a) Ti_3_AlC_2_ MAX‐phase raw particles; b) etched multilayered MXenes; representative TEM images of the obtained few‐layered Ti_3_C_2_T*_x_* MXene flakes c) at low magnification and d) at high magnification (the inset shows the corresponding SAED pattern); e) XRD patterns of Ti_3_AlC_2_ MAX‐phase raw particles and few‐layered Ti_3_C_2_T*_x_* MXene flakes; f) the colloidal solution containing few‐layered Ti_3_C_2_T*_x_* MXene flakes; g) a photo of the colloidal solution containing 1D BC fibers; the h) SEM image and i) TEM image corresponding to the 1D BC fibers.

**Figure 3:**
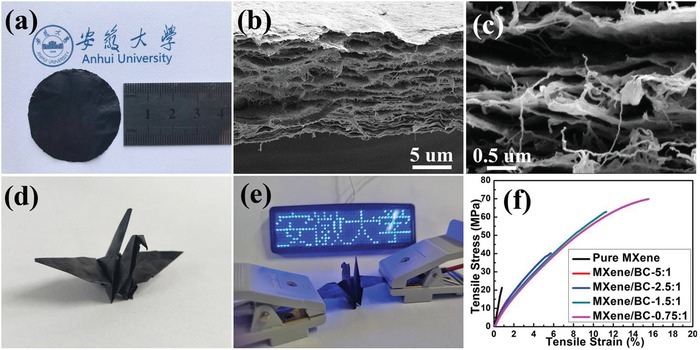
a) A photograph of a representative as‐prepared MXene/BC composite paper with a mass ratio of 1.5:1; b) the corresponding cross‐sectional SEM image and c) the magnified image of the MXene/BC composite paper showing the evenly distributed 1D BC fibers inserted between the layered Ti_3_C_2_T*_x_* sheets; d) a photograph of a paper crane folded from the as‐obtained MXene/BC composite paper with a mass ratio of 1.5:1; e) a photograph of paper crane serving as a conductor to light an LED; f) typical stress–strain curves of the pure MXene paper and MXene/BC composite papers prepared with mass ratios ranging from 5:1 to 0.75:1.

A schematic of the fabrication strategy for the stretchable all‐solid‐state planar MSCAs is shown in **Figure** **1**, which begins with the preparation of flexible and lightweight MXene/BC composite papers (Figure 1c) with excellent mechanical strength and electrochemical performance via a facile all‐solution‐based paper‐making process (Figure 1b). The MXene/BC composite papers have a layered structure consisting of restacked 2D fully delaminated few‐layered Ti_3_C_2_T*_x_* MXene flakes interspaced by 1D BC fibers. Large quantities of the 2D MXene flakes, with lateral sizes of ≈0.5–1.0 µm (**Figure** **2**c,d), were exfoliated by sonicating multilayered Ti_3_AlC_2_ powders (Figure 2b) obtained from acid etching Ti_3_AlC_2_ MAX‐phase raw powders (Figure 2a) according to a previously reported method.^51^, ^52^ The selective area electron diffraction (SAED) pattern (inset of Figure 2d) shows the hexagonal symmetry and single‐crystal nature of the flakes.^44^ Figure 2e shows the X‐ray diffraction (XRD) patterns of the Ti_3_AlC_2_ MAX‐phase raw powder and as‐prepared 2D few‐layered Ti_3_C_2_T*_x_* flakes. Notably, the strong characteristic peak of the Ti_3_AlC_2_ MAX‐phase at a 2θ value of ≈39° disappeared. Additionally, the (002) peak shifted from ≈10° to ≈6°, and the corresponding peak intensity was stronger, indicating the successful preparation of 2D few‐layered Ti_3_C_2_T*_x_* flakes.^54^ Herein, by virtue of the well‐known preparation conditions and excellent intrinsic properties (high volumetric capacitance of ≈1000 F cm^−3^ and conductivity of 6500 S cm^−1^), Ti_3_C_2_T*_x_* was chosen to fabricate the MXene/BC composite papers demonstrated in our work.^53^, ^54^ Additionally, the Ti_3_C_2_T*_x_* surfaces are terminated by functional groups such as —O, —OH, and —F. These negatively charged surface groups (primarily —OH and —O) endow the MXene sheets with excellent hydrophilicity and result in the formation of stable colloidal solutions, as shown in Figure 2f. Commercially available bacterial cellulose (BC) is an organic compound composed of d‐glucose coupled via β‐(1‐4)‐glycosidic linkages that is nearly inexhaustible and has outstanding physical and chemical stability.^62^ Importantly, BC possesses excellent mechanical properties with a high Young's modulus of 15–35 GPa and tensile strength of 200–300 GPa, which far outweigh those of other organic layered materials, such as polypropylene (Young's modulus of 1–1.5 GPa, tensile strength of 30–40 GPa), polyethylene terephthalate (Young's modulus of 3–4 GPa, tensile strength of 50–70 GPa), and cellophane (Young's modulus of 2–3 GPa, tensile strength of 20–100 GPa), making it suitable for use as a reinforcing additive in the fabrication of paper with enhanced mechanical strength.^63^ Furthermore, the electrostatic repulsion forces derived from the abundant surface functional groups such as —COOH and —OH, enable BC to be dispersed well in water without agglomeration (Figure 2g). After mixing the MXene colloidal solution and BC colloidal solution, a uniform hybrid suspension was obtained, as shown in Figure S1 in the Supporting Information. We found that after standing for 24 h, no obvious precipitation appeared in the hybrid solution consisting of colloidally dispersed MXene sheets and BC fibers, showing the good stability of the hybrid suspension, which is critical for obtaining a uniform freestanding defect‐free composite film through the all‐solution‐based paper‐making process. The good dispersity was also important for achieving both excellent mechanical strength and electron conductivity. However, it should be noted that after 24 h of standing, some aggregates were observed in the suspension. Based on this phenomenon and the results of previous reports,^64^, ^65^ we propose that hydrogen bonds are gradually generated between the surface functional groups of the colloidal BC fibers and MXene sheets during long‐term storage, which leads to the destruction of the force balance between neighboring colloidal particles due to neutralization of the charged surface groups, resulting in the formation of the observed aggregates, which consist of MXene sheets and BC fibers. Figure 2h,i shows that the BC fibers have a high aspect ratio with an average diameter and length of 50–100 nm and 20 µm, respectively. The high aspect ratio of the BC fibers was also favorable for their tight and stable wrapping of the supporting MXene sheets. Thus, the MXene/BC composite paper was easily fabricated through vacuum‐assisted filtration of a uniform suspension containing the well‐dispersed Ti_3_C_2_T*_x_* flakes and BC fibers (Figure 1a,b). The as‐fabricated MXene/BC composite paper (with a mass ratio of 1.5:1) was easily detached from the filter membranes, resulting in a free‐standing paper (**Figure** **3**a), with a thickness of ≈13.3 µm (Figure 3b). A freestanding active electrode film with excellent mechanical strength and electron conductivity is important for advanced stretchable and flexible electronics. Figure 3b shows a typical cross‐sectional scanning electron microscopy (SEM) image of the MXene/BC‐1.5:1 composite paper, in which a large number of 1D BC fibers evenly interspersed between the restacked 2D MXene sheets can be observed. In the design of this composite structure, the tightly packed 2D MXene sheets provide a highly efficient electron transport path, while the 1D BC fibers can greatly enhance the mechanical strength of the composite paper by functioning as a glue linking the 2D Ti_3_C_2_T*_x_* sheets together, as shown in the enlarged cross‐sectional image (Figure 3c). X‐ray photoelectron spectroscopy (XPS) was further employed to study the surface chemical environment of the as‐obtained MXene/BC‐1.5:1 composite paper, which indicated the presence of C, Ti, O, and F, and proved that the MXene surfaces are terminated by —O, —OH, and —F groups (Figure S2, Supporting Information). In addition, there were no new peaks in the XPS spectrum of the MXene/BC‐1.5:1 composite paper in comparison with that of pure MXene paper, indicating no chemical bonds formed between the MXene sheets and BC fibers. Thus, we believe that the interactions, between MXene sheets and BC fibers in the composite paper, were physical, mainly van der Waals forces and intermolecular hydrogen bonds generated between the abundant negatively charged surface groups (—O, —OH and —COOH, —OH) of the 2D MXene sheets and BC fibers. As expected, the as‐obtained composite paper could be freely rubbed, twisted, and folded (Video S1, Supporting Information), and even folded into a paper crane (Figure 3d), which served as a conductor to light an LED (Figure 3f), without noticeable damage to its structure, indicating its excellent flexibility, durability, and conductivity. In contrast, the as‐fabricated pure MXene paper without the addition of the 1D BC fibers was liable to break under slight bending, rubbing, or twisting (Video S2, Supporting Information).

To comprehensively and accurately evaluate the influence of the BC content on the reinforcement of the mechanical strength of the MXene/BC composite papers, a pure MXene paper and MXene/BC composite papers with mass ratios of 5:1, 2.5:1, and 0.75:1 were fabricated, as shown in Figure S3 in the Supporting Information, and their corresponding tensile tests were subsequently conducted. As shown in the as‐obtained stress–strain curves (Fig. 3f), the pure MXene paper exhibits a weak tensile strength of 21.3 MPa with a low strain at break of ≈0.8%, thus easily suffering from damage during the processes of stretching, twisting, and folding, as proven in Video S2 in the Supporting Information, which directly indicates the brittleness of the pure MXene paper. In contrast, incorporation of 1D BC fibers into 2D MXene sheets could effectively enhance the tensile strength of the composite papers. As shown in Figure 3f, with increasing mass fraction of the added 1D BC fibers, the tensile strengths of the MXene/BC composite papers were greatly improved. As a result, a tensile strength of up to 70.0 MPa (a strain at break of ≈15.5%) was achieved for the composite film prepared with a mass ratio of 0.75:1 (≈57 wt% BC), which represents an increase of more than three times that of the pure MXene paper, directly showing the effectiveness of the 1D BC fibers as an excellent reinforcing additive for the fabrication of MXene‐based flexible electrode films with enhanced mechanical strengths.

### 2.2 Standardized MSC Units: Fabrication and Electrochemical Performance

**Figure 4:**
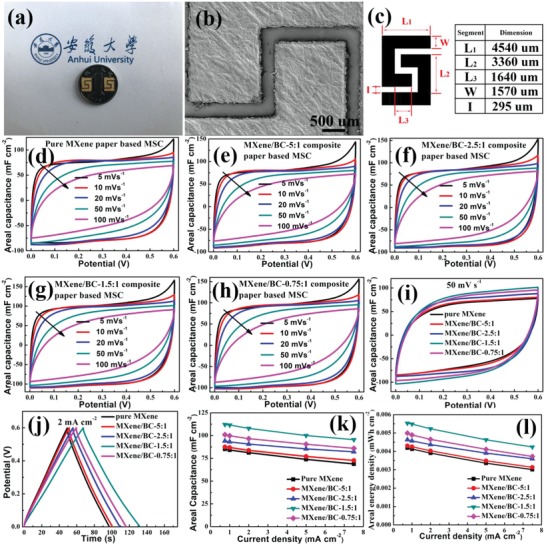
a) A photograph of the fabricated standardized MSC units based on the MXene/BC composite paper; b) corresponding SEM image of the symmetrical interdigitated electrodes of the MSC units; c) the geometric parameters of the electrodes CV curves of the fabricated standardized MSC units based on the MXene/BC composite papers prepared with mass ratios ranging from 5:1 to 0.75:1 and pure MXene paper at scan rates from 5 to 100 mV s^−1^: d) pure MXene paper; e) MXene/BC‐5:1 composite paper; f) MXene/BC‐2.5:1 composite paper, g) MXene/BC‐1.5:1 composite paper, and h) MXene/BC‐0.75:1 composite paper; i) CV curves of the different devices at a same scan rate of 50 mV s^−1^; j) GCD curves of the devices at a current density of 2 mA cm^−2^; evolution of the k) areal capacitance and l) areal energy density of the devices versus current density.

To further characterize the impact of the 1D BC reinforcing material on the electrochemical performance of the MXene/BC composite papers, as‐prepared MXene/BC composite papers with different mass fractions of the 1D BC fibers were further fabricated into standardized planar micro‐supercapacitor units (interdigital configuration with two positive electrodes and two negative electrodes as shown in **Figure** **4**a) by employing a simple and low‐cost laser‐cutting technique according to our previous report,^51^ and then tested with cyclic voltammetry (CV) and galvanostatic charge–discharge (GCD) methods. Prior to testing, a thin Au layer with a thickness of ≈80 nm was magnetron sputtered onto the surface of the MXene/BC composite papers, serving as the current collector and to improve the conductivity of the electrodes (Figure 1d). The active area of the as‐fabricated standardized MSC units was ≈0.3 cm^2^, as shown in Figure 4b,c, including an interelectrode spacing of ≈295 µm, an electrode width of 1570 µm, and a total electrode length of 9540 µm for both the positive or negative poles. The areal mass loading of the active MXene active material for each MSC unit was ≈2.0 mg cm^−2^. Figure 4d–i shows the CV curves of the as‐fabricated standardized MSC units (Figure 4a) based on pure MXene papers and MXene/BC composite papers with different mass fractions of the 1D BC fibers over the potential window of 0–0.6 V at different scan rates. All the curves exhibit good rectangular consistency, revealing the excellent conductivity of the electrodes and capacitive behavior of these MSC units. Interestingly, as the mass fraction of the 1D BC fibers increased, the peak current density of the corresponding MSC units showed an obvious increase for the same sweep rate, except for that based on the MXene/BC‐0.75:1 composite paper (Figure 4i). Moreover, as shown in the GCD curves of the as‐fabricated standard MSC units measured at the same current charge–discharge density (Figure 4j), the running time of the MSCs based on the MXene/BC composite papers showed an obvious increase with increasing mass fraction of the 1D BC fibers except for that based on the MXene/BC‐0.75:1 composite paper, directly indicating an enhanced areal capacitance of the MSC units based on MXene/BC composite papers with higher mass fractions of the 1D BC fibers, without the abnormal sample based on the MXene/BC‐0.75:1 composite paper being considered for the time being.

The available areal capacitance (mF cm^−2^) and energy density (mWh cm^−2^) provided per standardized MSC unit could be calculated from the GCD curves (Figure S4a–e, Supporting Information) according to the equations listed in the Experimental Section in the Supporting Information. Figure 4k,l compares the areal performance metrics of the standardized MSC units based on the pure MXene paper and the MXene/BC composite papers with different mass fractions of the 1D BC fibers. As shown, both the areal capacitance and energy density of the devices showed obvious growth, as expected from the addition of the 1D BC fibers. A maximal areal capacitance of 112.2 mF cm^−2^ and energy density of 0.00554 mWh cm^−2^ were achieved for the MSC unit based on the MXene/BC composite paper prepared with a mass ratio of 1.5:1, for which both values represent 132% enhancement compared to those based on pure MXene paper (85.13 mF cm^−2^ and 0.00419 mWh cm^−2^). In addition, these areal performance metrics are superior to those of the few currently reported MXene‐based planar MSCs (0.075–71.2 mF cm^−2^ and 0.00001–0.00352 mWh cm^−2^).^51^, ^52^, ^53^, ^54^, ^55^ In particular, the areal capacitance is an order of magnitude higher than that of carbon material–based planar MSCs (0.1–10 mF cm^−2^).^66^, ^67^, ^68^, ^69^, ^70^, ^71^, ^72^

The great improvement in the areal performance metrics is believed to be mainly due to the following reasons: First and foremost, employing few‐layered Ti_3_C_2_T*_x_* flakes with a high volumetric capacitance and conductivity to replace conventional carbon materials in the fabricated electrodes can substantially enhance the stored capacitance per unit of electrode area. Second, the laser‐cutting technique allows a minimal interelectrode spacing to be achieved between the interdigitated electrodes, which further ensures a short ion transport distance, resulting in a small electrolyte resistance. Third, the added 1D BC fibers can not only enhance the mechanical strength of the MXene/BC composite paper, but also effectively increase the interspatial distance between the 2D MXene flakes (c‐lattice parameter (c‐LP)) in the thick MXene/BC composite film electrodes, enabling full utilization of the surfaces of the 2D MXene flakes and exposure of a greater number of active sites for the electrolyte.

**Figure 5:**
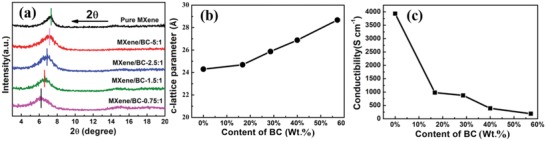
a) XRD patterns of the as‐prepared pure MXene paper and MXene/BC composite papers with mass ratios ranging from 5:1 to 0.75:1, which shows a decrease in the 2θ angle corresponding to the increased interspatial distance between the Ti_3_C_2_T*_x_* flakes of the paper; b) the c‐lattice parameter of the as‐fabricated MXene/BC composite papers versus the 1D BC fibers content; c) electrical conductivity versus the 1D BC fibers content in the MXene/BC composite papers.

As shown in the X‐ray diffraction patterns of the pure MXene paper and the as‐fabricated MXene/BC composite papers physically intercalated with different mass ratios of the 1D BC fibers (**Figure** **5**a), the (002) peak of the pure MXene paper shifted to lower 2θ angles with increasing weight content of the added 1D BC fibers. This indicated that the c‐lattice parameter of the MXene/BC composite papers intercalated with 1D BC slightly increased compared to that of pure MXene paper. Figure 5b shows the c‐LP in Å versus the weight content of the 1D BC fibers. As shown, the initial c‐LP of the pure MXene paper was calculated as 24.30 Å. After the addition of 1D BC fibers, the c‐LP gradually increased to 28.67 Å for MXene/BC‐0.75:1. This small increase in the c‐LP could not be attributed to the 1D BC fibers intercalated between the MXene sheets. However, all of the above results indicate that the added BC fibers served not only as anchors to link the MXene sheets together and enhance the mechanical strength of the as‐obtained composite MXene/BC paper, but also as interlayer nanospacers inserted between the 2D MXene flakes to reduce the restacking of the fully delaminated few‐layered MXene flakes during paper production, resulting in enhanced areal performance metrics attributed to the increased interlayer spacing between the 2D MXene flakes, which maximized accessibility of the electrolyte ions to the active electrode materials.

It should be noted that 1D carbon nanofibers have good conductivity in general. The addition of 1D carbon nanofibers will not lead to a significant attenuation of the electrical conductivity of the MXene/carbon nanofibers composite films. Unlike 1D carbon nanofibers, BC is an insulating material. The incorporation of the insulating 1D BC fibers between the conductive 2D MXene flakes will inevitably lead to an increase in the insulating contact area between the restacked 2D MXene flakes, which in turn negatively effects the transmission of electrons along the *c*‐axis of the restacked 2D MXene flakes, leading to synchronous conductivity attenuation with increasing weight content of the 1D BC fibers. The relationship between the electrical conductivity of the MXene/BC composite papers and the BC weight percentage is shown in Figure 5c. A high electrical conductivity favors electron transport and charge transfer inside thick‐film electrodes, guaranteeing the ideal electrochemical performance of the subsequently fabricated MSCs. Evidently, accompanied by the increase in the weight content of the added highly resistive 1D BC fibers, the corresponding electrical conductivity decreased gradually from 3934.3 S cm^−1^ for pure MXene paper to 190.2 S cm^−1^ for the MXene/BC‐0.75:1 composite paper. As a result, the improvement in the electrochemical performance derived from the increase of the c‐LP between the conducting 2D MXene flakes will be partially offset by the simultaneously emerging decrease in the electrochemical performance induced by the decreased electrical conductivity of the thick‐film electrodes. This may be the reason why the MXene/BC composite paper with excessive mass fraction of added 1D BC fibers (e.g., 0.75:1) showed a decreased electrochemical performance compared to that of the MXene/BC‐1.5:1 composite paper. However, we are optimistic that there may be room for further enhancement of the areal performance metrics of devices based on MXene/BC‐1.5:1 composite paper, possibly through anchoring conductive polymers such as polyaniline or polypyrrole onto the surface of the 1D BC fibers,^73^, ^74^, ^75^ which could reduce the conductivity decline by providing additional conductive pathways in the polymer shell surrounding the core of 1D BC fibers, improving the electron transport between the 2D MXene flakes isolated by the inserted 1D BC fibers, and contributing additional capacitance, thus serving as an active material.

### 2.3 MSCAs: Fabrication and Mechanical and Electrochemical Properties

Based on the above results, MXene/BC composite papers with a well‐balanced mass fraction of 1.5:1 were chosen as a trade‐off between the mechanical strength and areal performance metrics, guaranteeing the excellent performance of subsequently fabricated deformable all‐solid‐state MSCAs. To ensure the highly stretchable characteristics of the MSCAs, the key lies in the construction of highly elastic/stretchable conductive frameworks (bridges) interconnecting the MSC islands. To date, to obtain high elongation, the few reports on stretchable MSCAs mainly employed serpentine metallic interconnections fabricated via a photolithography process or embedded liquid metal interconnections such as galinstan as the elastic/stretchable conductive framework to interconnect the MSC islands.^33^, ^34^, ^35^, ^36^, ^37^, ^38^, ^39^ Both the photolithography and liquid metal process will result in low production efficiencies and high costs for the MSCA devices, which would be very detrimental to the large‐scale production of MSCA devices and their commercial application. Thus, exploring a simple, low‐cost, and high‐efficiency method to realize the construction of a highly elastic/stretchable conductive framework that does not involve complicated and tedious processes or expensive equipment and materials is of great significance for the development of stretchable MSCAs with high elongation factors. Laser‐cutting is a pattern‐on‐demand technique that allows the computerized patterns to be formed in a single step on various substrates. Herein, by adopting the laser‐cutting technique, based on the as‐prepared MXene/BC composite paper, integrated modularized MSC units in an arbitrarily connected in series or in parallel via a highly stretchable conductive framework (bridges) can be easily fabricated in a single step without involving conventional photolithography processes or using any external liquid metal wires (Figure 1e), which greatly simplifies the whole fabrication process, enhances the production efficiency, and reduces the cost. More importantly, in the segmented structural design, the MXene/BC composite paper with an interdigital pattern (Figure 1f) only served as modularized MSC islands for energy storage, which were subjected to a near‐zero amount of strain because of the nearly rigid‐body motion of their strain‐suppressed zone during stretching, thus guaranteeing the excellent electrochemical stability of the MSCA device. Additionally, the MXene/BC composite paper with a long strip pattern served as the stretchable conductive framework (bridges) to interconnect these modularized MSC islands, forming an island–bridge structure and ensuring the high stretchability of the MSCA device. The integrated modularized MSC units with an island–bridge structure can function as a stretchable MSCA device with a high elongation factor and electrochemical stability (Figure 1h). For the purpose of supporting the patterned electrodes and further enhancing the robustness of the as‐fabricated MSCAs and prevent their being torn when the devices suffer from rough stretching, twisting, and bending, an identically sized ≈0.03 mm thick double‐sided adhesive tape layer (Figure 1g; Figure S5, Supporting Information) was used to bond the patterned MXene/BC composite electrodes to the flexible PET film support with a similar island–bridge structure, which favorably improved the deformational stability. Finally, a PVA/H_2_SO_4_ gel electrolyte (Figure 1g) was drop‐cast onto the MSC islands for ionic transport. A schematic diagram and a representative SEM image of the multilayered structure of the MSCAs are shown in Figure 1g and Figure S5 in the Supporting Information, respectively. A detailed description of the fabrication process is given in the Experimental Section in the Supporting Information).

**Figure 6:**
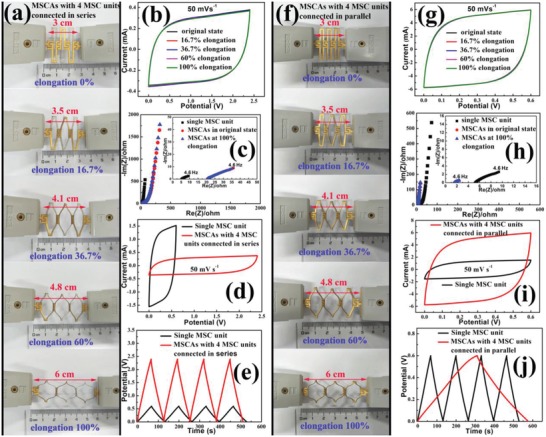
a) Optical photographs of an as‐fabricated MSCA consisting of 4 MSC units connected in series under an applied strain ranging from 0% to 100% elongation, b) corresponding CV curves, c) Nyquist plots for a single MSC unit and the MSCA before and after application of a 100% elongation strain (inset shows the curve enlarged in a high‐frequency region), d) CV curves for the MSCA and a single MSC unit at a scan rate of 50 mV s^−1^, and e) GCD curves for the MSCA and a single MSC unit at a high current density of 2.0 mA cm^−2^. f) Optical photographs of an as‐fabricated MSCA consisting of 4 MSC units connected in parallel under an applied strain ranging from 0% to 100% elongation, g) corresponding CV curves, h) Nyquist plots for a single MSC unit and the MSCA before and after application of a 100% elongation strain (inset shows the curve enlarged in the high‐frequency region), i) CV curves for the MSCA and a single MSC unit at a scan rate of 50 mV s^−1^,and j) GCD curves for the MSCA and a single MSC unit at a high current density of 2.0 mA cm^−2^.

**Figure** **6**a,f shows typical photographs of as‐fabricated MSCAs consisting of 4 MSC units connected in series or in parallel. Thanks to the employed laser‐cutting technique, MSCAs with a segmented electrode structural design can be fabricated in a single step, which greatly simplifies the fabrication process. As expected, benefiting from the island–bridge interconnected structural layout, the as‐fabricated MSCAs exhibited high stretchability and could be smoothly and reversibly stretched between 0% and 100% elongation, with a nearly linear response to the applied tension. More importantly, no obvious deterioration was detected in the in situ recorded CV curves (Figure 6b,g), demonstrating an equally excellent electrochemical stability. The unnoticeable change in the acquired equivalent series resistance (ESR) of the MSCAs before (20.57 Ω for the in series configuration, and 1.48 Ω for the in parallel configuration) and after (20.66 Ω for the in series configuration and 1.49 Ω for the in parallel configuration) the application of a 100% elongation strain also indirectly proves the presented conclusion, as shown in the insets of Figure 6c,h. In addition, the segmented structural design, which allows the modularized MSC units to be arbitrarily grouped and interconnected by the elastic/stretchable bridges, enables on‐demand control of the output voltage and current density of the MSCAs to meet actual requirements. Figure 6d shows the CV curves obtained from a single MSC unit and the MSCA consisting of 4 MSC units connected in series at a scan rate of 50 mV s^−1^. It can be seen that the output voltage of the MSCA has increased to four times that of the single modularized MSC unit, while the discharge time was approximately equal, as further shown in Figure 6e. This indicated that MSCAs fabricated by assembling modularized MSC units in series can output a multiplied voltage. The obtained CV curves of a single modularized MSC unit and the 4 MSC units connected in parallel are shown in Figure 6i (sweep rate: 50 mV s^−1^, voltage range: 0–0.6 V), in which the output current of the MSCA is close to quadrupled that of the single modularized MSC unit. In addition, the runtime of the MSCA is also quadruple the value of the single MSC unit at the same current density of 2.0 mA cm^−2^, as shown in the GCD curves (Figure 6j), from which the calculated capacitances for the MSCA and the single modularized MSC unit are 133.8 and 32.4 mF, respectively. This indicates that the output current and delivered capacitance can be easily increased through the fabrication of MSCAs consisting of modularized MSC units connected in parallel. Therefore, it can be safely concluded that the simplified laser‐cutting fabrication of the MSCAs with on‐demand arrangement of MSC units connected in parallel or in series can not only ensure a high stretchability and electrochemical stability of the MSCAs, but also realize a controllable increase in output voltage and current density to meet actual requirements, making this protocol a powerful strategy for the development of MXene‐based planar MSCAs with high areal performance metrics, stretchability, and electrochemical stability. A demo video, which was recorded continuously as an MSCA was stretched from 0% to 100% elongation, is shown in Video S3 in the Supporting Information. As shown, the planar MSCA consisting of 4 MSC units connected in series was stretched and deformed smoothly and could light a commercial LED during the process, directly demonstrating the great potential of the as‐fabricated MSCAs for use as deformable micropower sources in flexible/stretchable electronic devices.

**Figure 7:**
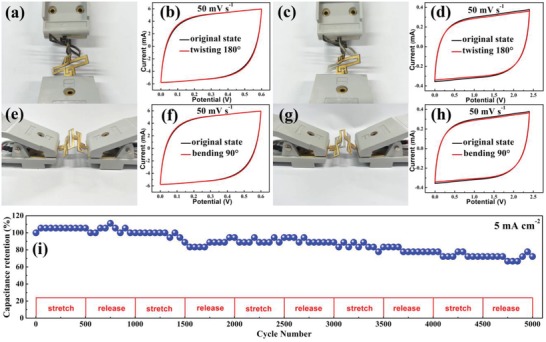
Photographs of an as‐fabricated MSCA containing 4 MSC units connected in parallel when a) twisted to 180° and e) bent to 90°, and b,f) the corresponding CV curves; photographs of an as‐fabricated MSCA containing 4 MSC units connected in series when c) twisted to 180° and g) bent to 90°, and d,h) the corresponding CV curves; i) capacitance retention of an as‐fabricated MSCA containing 4 MSC units connected in series during repeated tensile deformation between 0% and 100% elongation over 5000 cycles.

Notably, the as‐fabricated all‐solid‐state planar MSCAs also exhibited excellent twistability, bendability, and good cycling stability in addition to the stretchability. As shown in **Figure** **7**a,c,e,g, the island–bridge interconnected structure enabled the MSCAs to easily realize out‐of‐plane deformations to accommodate the exerted bending and torsional stresses, while the in situ recorded CV curves obtained under bending to 90° and twisting to 180° fully overlapped with those obtained in the normal state at the same scan rate (Figure 7b,d,f,h), proving the excellent twistability and bendability of the MSCA devices. Cyclic stability is one of the most important requirements when a new energy storage system is applied. To further verify the cyclic stability of the MSCAs, a MSCA device consisting of 4 MSC units connected in series was stretched/released every 500 cycles under an applied 100% elongation strain for 5000 cycles, and a GCD test at a constant current density of 5 mA cm^−2^ was carried out to record the results in situ. Thanks to the segmented structural design, in which the modularized MSC units do not experience tensile deformation and are only responsible for energy storage, the MSCAs are not vulnerable to destruction under repeated tensile deformation, guaranteeing the electrochemical stability of the devices. Thus, as demonstrated in the results of the cycle performance (Figure 7i), the devices can retain 72.2% of their initial capacitance after 5000 cycles under repeated tensile deformation between 0% and 100% elongation, indicating the good stability of the capacitive charge–discharge behavior of the stretchable devices. For a more intuitive understanding of the performance of the demonstrated MSCAs, which were fabricated through the combination of high‐performance MXene/BC composite paper and a laser‐cutting kirigami patterning technique, the areal performance metrics and elongation of the devices are compared with those of previously reported state‐of‐the‐art MSCAs in **Table** **1**. As shown in Table 1, it can be seen that the MSCAs consisting of 4 MSC units connected either in parallel or in series exhibited outstanding areal performance metrics with high areal capacitances of 111.5 or 6.61 mF cm^−2^, respectively, at a current density of 2 mA cm^−2^, as well as a high elongation of 100%, powerfully demonstrating the effectiveness of this protocol for fabricating high‐performance MSCAs for use as deformable micropower sources.

**Table 1:** List of the areal capacitances and elongations of recently reported state‐of‐the‐art MSCAs, our demonstrated MSCAs, and stretchable MSCs in the conventional wave‐like configuration

Electrode material	Areal capacitance [mF cm^−2^]	Elongation [%]	Ref.
Few‐layered MXene flakes	111.5/6.61 (Parallel/series connection)	100	This work
Polypyrrole/CNT	5.17	30	34
SWCNTs	0.74	30	28
SWCNTs	0.000042	150–275	14
MWCNT/Mn_3_O_4_	0.57	40	38
MWCNTs	0.000041	50	39
MWCNTs	0.51	80	37
MWCNT/Mn_3_O_4_	0.4895	50	36
rGO (MSC in the conventional wave‐like configuration, single unit)	0.54	100	32
MWCNT/PANI (MSC in the conventional wave‐like configuration, single unit)	44.13	40	29

## 3 Conclusion

In summary, through the combination of strain engineering and modulation of the interlayer spacing, a freestanding and lightweight MXene/BC composite paper with excellent mechanical strength and electrochemical performance was designed and fabricated via a facile all‐solution‐based paper‐making method. Following an efficient laser‐cutting kirigami patterning process, stretchable, bendable, and twistable all‐solid‐state micro‐supercapacitor arrays were further fabricated. Benefiting from the high‐performance MXene/BC composite electrodes and rational sectional structural design, the resulting deformable MSCA exhibited not only outstanding areal performance metrics with a high areal capacitance of 111.5 mF cm^−2^ and a high areal energy density of 0.00552 mWh cm^−2^, but also high electrochemical stability under dynamic stretching/relaxing, and in bent or twisted states. More importantly, the rational geometrical design of the kirigami patterning allows the modularized MSC islands to be arbitrarily grouped and interconnected by a stretchable conductive framework, enabling on‐demand control of the output voltage and current density to meet actual requirements. The demonstrated combination of a high‐performance MXene/BC paper‐making method with a simple laser‐cutting kirigami patterning technique provides a promising approach for designing and fabricating high‐performance MXene‐based deformable MSCA devices for use as micropower sources that are compatible with wearable microelectronic devices.

## Conflict of Interest

The authors declare no conflict of interest.

## Supporting information

Supplementary
