# Supplementary material for: Kirigami Patterning of MXene/Bacterial Cellulose Composite Paper for All‐Solid‐State Stretchable Micro‐Supercapacitor Arrays
Source: Adv Sci (Weinh). 2019 Apr 12;6(12):1900529. doi: 10.1002/advs.201900529 (PMC6662096; doi:10.1002/advs.201900529)
Supplement: Supplementary file 1 — Supplementary [file ADVS-6-1900529-s001.pdf]

Copyright WILEY-VCH Verlag GmbH & Co. KGaA, 69469 Weinheim, Germany, 2019.

ADVANCED SCIENCE
Open Access

Supporting Information

for *Adv. Sci.,* DOI: 10.1002/advs.201900529

Kirigami Patterning of MXene/Bacterial Cellulose Composite Paper for All-Solid-State Stretchable Micro-Supercapacitor Arrays

Shangqing Jiao, Aiguo Zhou, Mingzai Wu, and Haibo Hu**

Supporting Information

Kirigami patterning of MXene/bacterial cellulose composite paper for all-solid-state stretchable microsupercapacitor arrays

Shangqing Jiao, Aiguo Zhou, Mingzai Wu, and Haibo Hu**

Mr. S. Q. Jiao, Dr. M. Z. Wu, and Dr. H. B. Hu

School of Physics and Materials Science, Anhui University, Hefei, 230601, China

E-mail: haibohu@ahu.edu.cn; wumz@ahu.edu.cn

Dr. A. G. Zhou

School of Materials Science and Engineering, Henan Polytechnic University, Jiaozuo, 454000, China

Keywords: MXene, bacterial cellulose, kirigami, stretchable, micro-supercapacitor arrays

Experimental Section

Materials:

Hydrochloric acid (HCl), Sulphuric acid (H_2SO_4), Lithium fluoride (LiF), Polyvinyl alcohol (PVA-1750±50) were of analytic grade from the Sinopharm Chemical Reagent Co., Ltd. The bacterial cellulose (BC) was from Guilin Qihong Technology Co., Ltd. The MAX (Ti_3AlC_2, 400 mesh) was from 11 Technology Co., Ltd. The conductive silver paste was from Uniwell International. All reagents were used as received without further purification. Deionized water (18.4 MΩ cm^{-1}) was obtained with water purification machine (Jiangyin Company, Jiangsu Province).

Preparation of multi-layered Ti_3C_2Tx powders:

First, an etching solution consisting of 1.0 g LiF and 20 ml 6 M HCl was employed to extract the contained Al in the Ti_3AlC_2 MAX raw powders (1.0 g) under 35 °C for 24 h. Then, after centrifugation, removed the etching solution and washed the sediment with deionized (DI) water several times until the pH of the discarded upper liquid achieved 6. Finally, the multi-layered Ti_3C_2Tx powders were obtained after drying under normal atmospheric temperature.

Preparation of fully delaminated few-layered Ti_3C_2Tx flakes:

First, 0.2 g as-obtained multi-layered Ti_3C_2Tx powders were dispersed in 100 ml deionized water, followed by ultrasound exposure for 60 minutes in ice water bath. Then the unexploited multi-layered Ti_3C_2Tx powders were removed by centrifugation at 4000 rpm for 5 minutes. Finally, a colloidal solution containing fully delaminated few-layered Ti_3C_2Tx flakes (0.5 mg ml^{-1}) can be obtained.

Preparation of MXene/BC composite papers with different mass fraction of 1D BC fibers:

Typically, a variety of commercial BC (0.5 mg ml^{-1}) dispersions were controllably added to as-prepared MXene colloid solution (0.5 mg ml^{-1}, 50 ml) to prepare the composite inks with BC content from 0-57.1 wt% under stirring. Then, the MXene/BC composite papers were prepared by rapid filtration of the obtained ink in batches through a mixed cellulose ester membrane (47 mm in diameter, 0.45 um pore size, Whatman, Germany), which were finally peeled off from the filter paper and dried for 24 h. Pure MXene papers were also prepared with the same procedure.

Fabrication of standardized MSCs units based on MXene/BC composite papers:

First, interdigital-fingers-like circuit patterns were designed with AutoCAD software on a personal computer. Then, input the software into a laser cutting machine (TR-5030, professional CO_2 Universal Laser System, Shenzhen Triumph Industrial Co.,LTD). The laser power was set to 20% (10 W) and the speed was set to 200 mm s^{-1}, respectively. The Z-distance between the laser and the sample was 1.0 cm, while the laser beam size was about 100 μm. Before the laser-cutting process, a thin Au layer with thickness of about 80 nm was magnetron sputtered on the surface of the MXene/BC composite papers, served as the current collector and for better conductivity. Finally, with the aid of the laser cutting, the designed coplanar interdigital electrodes based on the MXene/BC composite papers can be fabricated. The polyvinyl alcohol (PVA)/sulfuric acid (H_2SO_4) gel electrolyte was used as the solid electrolytes, which was prepared through dissolving PVA powder (5 g) with H_2SO_4 aqueous solution (5 g H_2SO_4 into 50 ml DI water). The mixture was heated to 85 °C under vigorous stirring until the solution became clear. After cooling down, the gel solution was coated on the surface of interdigital electrodes. After the gel was solidified, the preparation of the standard MSCs based on MXene/BC composite papers was finished.

Fabrication of MSCAs based on MXene/BC composite papers:

MSCAs were also fabricated according to the above procedure. First, honeycomb patterns were designed with AutoCAD software on a personal computer. After the laser-cutting process, an identical ~0.03-mm-thick double faced adhesive tape is used to bond the patterned MXene/BC composite electrodes to the bottom flexible PET film supporter with the similar honeycomb structure, for the purpose of supporting the pattered electrodes and further enhancing the robustness of the as-fabricated MSCAs to prevent being torn when the devices suffer from rough stretching, twisting and bending, resulted in improved deformational stability. Finally, the PVA-H_2SO_4 gel electrolyte was drop-casting onto the MSC islands for ionic transport. A schematic diagram of the fabrication procedure of the MSCAs is shown in Figure 1.

The areal capacitance (Cs, mF cm^{-2}) and areal energy density (Ws, mWh cm^{-2}) of the MSCs and MSCAs were calculated from the charge-discharge curves according to the following equations:

$$C = \frac{Q}{\Delta E} = \frac{I\Delta t}{\Delta E} \quad (1)$$

$$Cs = \frac{C}{S} = \frac{I\Delta t}{S\Delta E} \quad (2)$$

$$Ws = \frac{0.5C(\Delta E)^2}{3600s} \quad (3)$$

where C is the total capacitance, Q is the total charge, I is the discharge current, Δt is the discharge time, ΔE is the potential window during the discharge process after *IR* drop, and S is the total area of the positive and the negative electrodes.

Tensile test and electrochemical measurements:
Tensile property of the MXene/BC composite paper samples of 3.0*1.0 cm (length*width) was characterized using a dynamic mechanical analysis (DMA) Q800 apparatus (TA Instruments Inc., U.S.) at room temperature. At least five specimens were used for each sample in the tensile test. Electrochemical properties of all the fabricated devices were investigated in a two-electrode configuration. Two Cu wires were connected to the pad of each microelectrode using Ag paste to make a connection to the electrochemical instruments. CV, EIS, and GCD measurements of MSCs and MSCAs were carried out on an electrochemical workstation (CHI 660E, Chenhua, Shanghai). Impedance spectroscopy measurements were performed at open circuit voltage with ±10 mV amplitude.

Material Characterization:
The micromorphology and phase composition of all samples were characterized by Field-emission scanning electron microscopy (FE-SEM, S-4800, Hitachi, Japan), Transmission electron microscopy (TEM, JEM-2100, JEOL, Japan), and X-ray powder diffraction (XRD Bruker D8-ADVANCE) with an 18 kW advanced X-ray diffrac-tometer with Cu K_{α} radiation (λ=1.54056 Å). Sheet resistance of vacuum-assisted deposited MXene-based film on paper was measured by a standard four-point probe method (RST-9, Four-Probe Tech.). A Nikon D3100 digital single lens reflex camera was employed to take all the optical pictures.

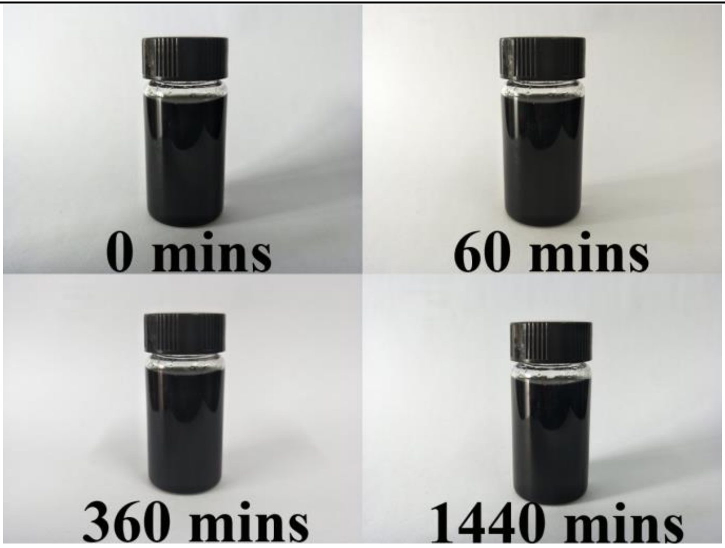

Figure S1. Optical photographs of a mixed solution of colloidal 2D Ti_3C_2Tx sheets and 1D BC fibers during continually standing for 24 h.

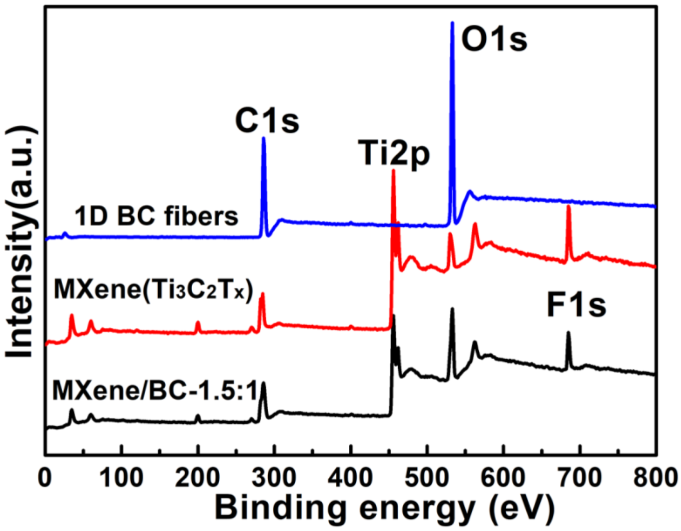

Figure S2. XPS survey spectrum of the pure BC fibers paper, pure MXene (Ti_3C_2Tx) paper, and MXene/BC-1.5:1 composite paper.

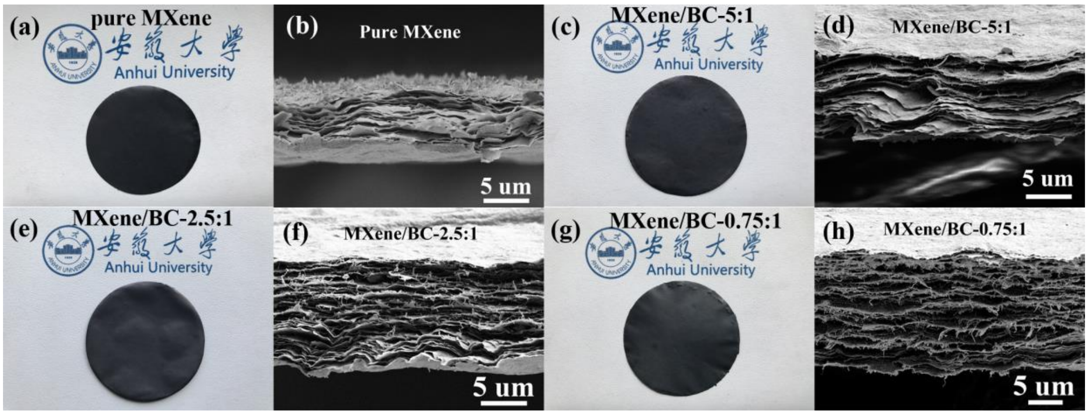

Figure S3. Typical photo graphs and corresponding cross-sectional SEM images of as-prepared MXene/BC composite papers with different mass fraction of the added 1D BC wires, a) and b) for pure MXene paper; c) and d) for MXene/BC-5:1 composite paper; e) and f) for MXene/BC-2.5:1 composite paper; g) and h) for MXene/BC-0.75:1 composite paper.

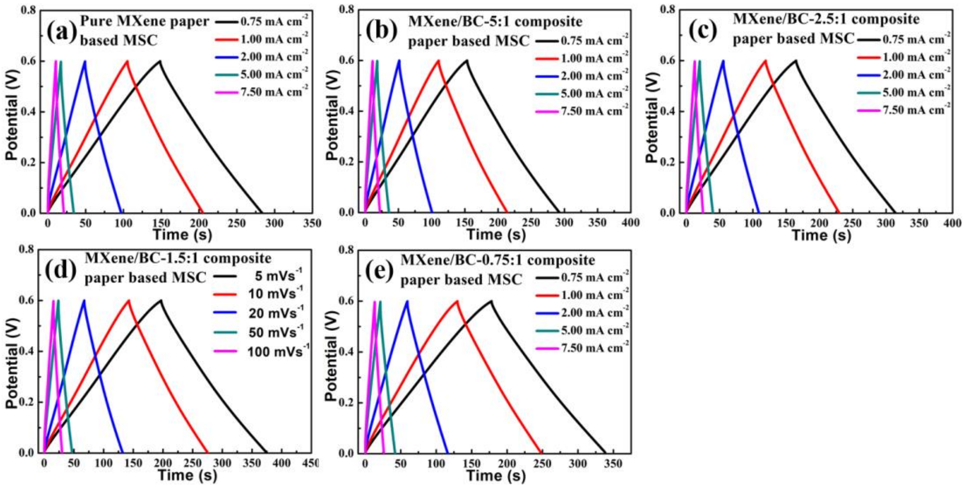

Figure S4. Galvanostatic charge-discharge curves of the as-prepared MXene/BC composite papers with different mass fraction of the added 1D BC wires, a) for pure MXene paper; b) for MXene/BC-5:1 composite paper; c) for MXene/BC-2.5:1 composite paper; d) for MXene/BC-1.5:1 composite paper; e) for MXene/BC-0.75:1 composite paper.

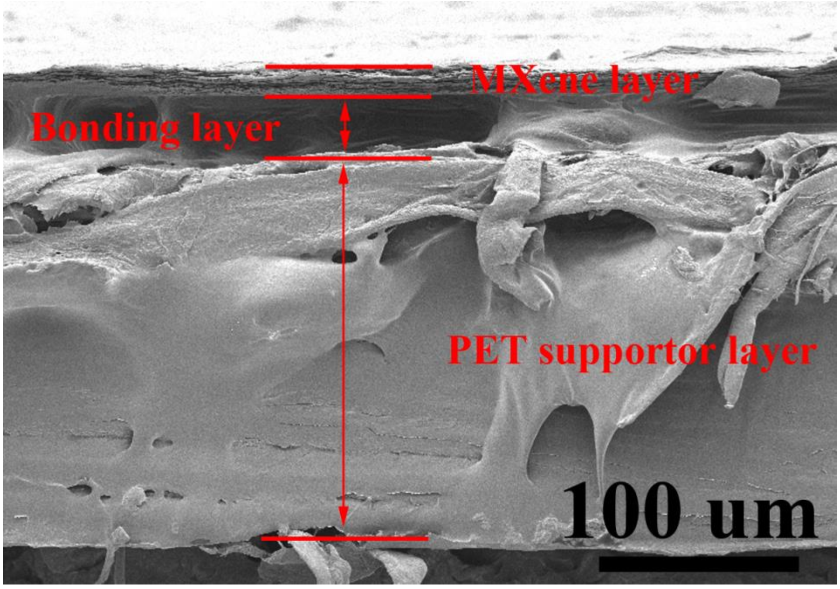

Figure S5. A representative SEM image of the multilayer structure of the MSCAs.
